# Hypothermia differentially modulates the formation and decay of NBS1, γH2AX and 53BP1 foci in U2OS cells exposed to gamma radiation

**DOI:** 10.1038/s41598-022-09829-y

**Published:** 2022-04-07

**Authors:** Magdalena Płódowska, Wiktoria Krakowiak, Aneta Węgierek-Ciuk, Anna Lankoff, Karol Szary, Krzysztof Lis, Andrzej Wojcik, Halina Lisowska

**Affiliations:** 1 Department of Medical Biology, Institute of Biology, Jan Kochanowski University, Kielce, Poland; 2 Centre for Radiobiology and Biological Dosimetry, Institute of Nuclear Chemistry and Technology, Warsaw, Poland; 3 Department of Atomic Physics and Nanophysics, Institute of Physics, Jan Kochanowski University, Kielce, Poland; 4 Department of Medical Physics, Holy Cross Cancer Center, Kielce, Poland; 5 Centre for Radiation Protection Research, Department of Molecular Biosciences, The Wenner-Gren Institute, Stockholm University, Stockholm, Sweden

**Keywords:** Cell division, DNA damage and repair

## Abstract

In studies on the mechanism of DNA damage response where ionizing radiation is used as the DNA damaging agent, cells are often exposed to ionizing radiation on melting ice (corresponding to 0.8 °C). The purpose of this procedure is to inhibit cellular processes i.e. DNA repair. Low temperature at exposure has been shown to act in a radioprotective manner at the level of cytogenetic damage, but its mechanisms of action are poorly understood. The aim of the study was to analyze the effect of hypothermia at the level of formation and decay of NBS1, γH2AX, and 53BP1 foci, micronuclei, survival, cell cycle progression and oxidative stress in U2OS cells. The results show that hypothermia alone induced oxidative stress and foci. When applied in combination with radiation but only during the exposure time, it potentiated the formation of γH2AX and 53BP1 but not of NBS1 foci. When applied during irradiation and subsequent repair time, 53BP1 and NBS1 foci formed and decayed, but the levels were markedly lower than when repair was carried out at 37 °C. The frequency of micronuclei was elevated in cells irradiated at 0.8 °C, but only when analysed 20 h after irradiation which is likely due to a reduced G_2_ cell cycle block. Hypothermia reduced cell survival, both with and without radiation exposure. The temperature effect should be considered when cooling cells on melting ice to inhibit DNA repair in the induction of DNA damage.

## Introduction

In studies aiming at better understanding the mechanisms of DNA damage response (DDR), ionising radiation is often used as the DNA damaging agent^1–3^. The reason for this is the precision of DNA damage induction, both in terms of the dose (which determines the damage level) and the time of damage delivery. A common approach is to irradiate cells on melting ice^4^. The rationale for this setup is to inhibit DNA repair during exposure and initiate it by transferring cells to 37 °C after irradiation. It is assumed that the only effect of cooling cells on melting ice is the reversible attenuation of DNA repair.

However, low temperature at exposure has been shown to act in a radioprotective manner at the level of cytogenetic damage, indicating that hypothermia at exposure not only inhibits DNA repair but also modulates the DDR^5^. The sparing effect of hypothermia on radiation-induced cytogenetic damage is referred to as the temperature effect (TE) and its mechanisms are neither well understood, nor has it been detected in all studies and in all cell lines. A review of earlier studies and of possible mechanisms of TE can be found in Brzozowska et al.^6^ and Dang et al.^7^.

In experiments with human peripheral blood lymphocytes (PBL) and the human lymphoblastoid cell line TK6, we observed the TE both as reduced levels of chromosomal aberrations and micronuclei (MN)^5–8^. However, TE was not visible at the level of γH2AX focus formation and decay^5,7^ or the alkaline and neutral comet assay^6^ suggesting that it results from the perturbation of the cell cycle transition or a modified transition of DNA damage into cytogenetic damage.

Cytogenetic damage is believed to result from misrepaired DNA double strand breaks (DSB)^9^ which is the most lethal type of DNA damage induced by ionising radiation^10^. The response of cells to DSB induction is a complex but orchestrated process involving the activation of sensors, transducers and effector proteins^11^. DSB signalling depends on the position of the cell in the cell cycle, but major players are ATM, ATR and DNA-PK^11^. ATM is the most versatile kinase and is recruited to DSBs by the MRE11–RAD50–NBS1 (MRN) complex, whereby NBS1 is its specific protein co-factor responsible for a stable recruitment of the complex to DNA damage sites. Once it binds to a DSB, ATM phosphorylates the histone H2AX leading to the activation of a signalling cascade that results in 53BP1 recruitment to the site of a DSB. 53BP1 is classified as an adaptor/mediator, required for processing of the DNA damage response signal and as a platform for recruitment of other repair factors. Chronologically, the activation of NBS1 precedes the phosphorylation of H2AX that is followed by the activation of 53PB1^11^.

It remains unclear which stages of DDR are influenced by hypothermia during irradiation, leading to modified levels of cytogenetic damage. In an attempt to shed more light on this aspect we carried out experiments with U2OS cells which permanently express either the NBS1 protein or the 53BP1 protein, both tagged with the fluorescence green protein (GFP), allowing the direct and instantaneous analysis of formation and decay of ionising radiation-induced foci (IRIF) involving each protein^12,13^. In order to complement the results observed at the level of NBS1 and 53BP1, γH2AX foci were also detected using immunolabelled monoclonal antibodies^14^. Cells were exposed to radiation at 37 °C or on melting ice, transferred thereafter to 37 °C and fixed for analysis of foci at successive times. The temperature of melting ice in our laboratory was determined as 0.8 °C^7^ and this value is used in the current report. In order to see if DNA repair at all takes place at 0.8 °C, the kinetics of focus formation and decay was analysed in cells kept on melting ice for the whole period of analysis. The focus frequencies were compared with levels of micronuclei in cells transferred to 37 °C post exposure representing the outcome of DNA damage misrepair. Micronuclei were scored in cells harvested after 20, 26 and 32 h of culture time in order to identify the possible impact of cell cycle perturbation on the level of cytogenetic damage. Oxidative stress in cells treated by hypothermia was quantified by the dihydrorhodamine 123 test. The results were complemented by the analysis of cell cycle progression and clonogenic cell survival.

## Materials and methods

### Cell culture

Experiments were carried out with human osteosarcoma U2OS cells which were stably transfected with a plasmid coding for either 53BP1-GFP or NBS1-GFP. These cells were constructed as described in Bekker-Jensen et al.^12^ and Lukas et al.^13^ and kindly provided by the authors. The culture medium was composed of Dulbecco Modified Eagle Medium (CORNING, cat. 10-014-CVR) supplemented with 10% bovine calf serum (Biowest, Fetal Bovine Serum Premium, cat. S181BH-500) and 400 µg/mL geneticin G418 (Sigma-Aldrich, AI720). A high level of 53BP1-GFP and NBS1-GFP expression was assured by culturing cells in a selection medium containing geneticin to eliminate cells that lost the phenotype. U2OS cells were kept in a 5% CO_2_ humidified 37 °C incubator. Cells were grown on round glass coverslips before irradiation.

### Irradiation

Prior to irradiation, exponentially growing NBS1-GFP and 53BP1-GFP cells were placed for 30 min in styrofoam-coated plastic boxes to maintain the respective irradiation temperature of 0.8 °C or 37 °C. For 0.8 °C the boxes contained melting ice, for 37 °C the boxes contained water at that temperature. Cells were plated in 6 cm diameter Petri dishes containing 5 ml of medium. The Petri dishes were placed on the melting ice or the warm water. Cells were irradiated at the respective temperature with 2 Gy of 6 MeV photons using a medical linear accelerator (Artiste, Siemens, Germany) at a dose rate of 1 Gy/min and a source-to-skin distance (SSD) of 1 m. After irradiation, cells were placed in a humidified 37 °C incubator with 5% CO_2_.

### *K*inetic of foci formation and decay

**Figure 1:**
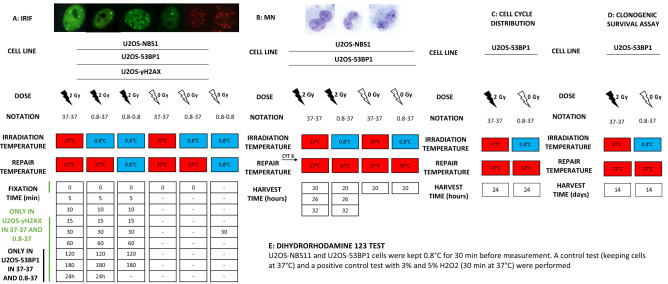
Scheme of the experimental setup to study IRIF (**A**), micronuclei (**B**) in U2OS-NBS1 and U2OS-53BP1 cells, distribution of cells in the cell cycle (**C**), clonogenic survival assay (**D**) and oxidative stress assay (**E**) in U2OS-53BP1 cells. Images show representative pictures of IRIF in U2OS-53BP1 cells (**A**, first two from left), U2OS-NBS1 cells (**A**, next two from left), U2OS-γH2AX cells (**A**, last two from left) and MN in U2OS (**B**) cells exposed to 2 Gy. IRIF after a repair time of 24 h were only analysed in U2OS-53BP1 and U2OS-γH2AX cells. *MN* micronuclei, *IRIF* ionising radiation-induced foci.

Kinetic of NBS1, 53BP1 and γH2AX foci formation and decay was analysed after 2 Gy of gamma radiation (Fig. 1A). NBS1 and 53BP1 foci were visualised thanks to GFP tagging. γH2AX foci were detected by immunostaining. No counterstaining of nuclei was applied. Cells were seeded on 22 × 22 mm glass coverslips and placed in 6 cm diameter Petri dishes containing 5 ml of medium. Cells were seeded at a density of 70,000/glass cover slip and exposed to radiation 48 h later as described above. After irradiation, cells were incubated to allow repair in a 5% CO_2_ humidified 37 °C incubator and fixed after 0, 5, 10, 15, 30, 60, 120, 180 min and 24 h. The fixation scheme varied according to cell line and experiment (Fig. 1A). Control cells were kept at 0.8 °C and 37 °C. For NBS1 and 53BP1 foci, cells on coverslips were fixed with 3% formaldehyde for 15 min at room temperature, washed with PBS and placed on cavity slides with wells filled with PBS. For γH2AX foci, cells on coverslips were fixed in 90% methanol for 5 min. Cells were permeabilised in blocking buffer (skimmed milk + bovine serum albumin (BSA/Sigma-Aldrich)) and incubated with anti-phospho-histone H2AX antibody Ser139 (Upstate/Millipore) followed by secondary anti-mouse IgG Alexa Fluor 546 (Invitrogen/Thermo Fisher), both in PBS containing 2% bovine serum albumin (BSA). Images of individual cells were taken at a single Z value using a confocal microscope (Nikon, A1+) and a 40 ×/1.25 WI lens. Constant and same settings for green laser intensity and image integration time were used for acquiring images of NBS1 and 53BP1. Constant settings for red laser intensity and image integration time were used for acquiring images of γH2AX foci. At least 40 cells per focus type and time point were analysed by manually counting foci on images.

A nested analysis was carried out on 53BP1 foci in cell nuclei corresponding to different phases of the cells cycle. The details of this analysis are described in Supplementary Material 1.

### Level of reactive oxygen species

Oxidative stress induced by hypothermia was quantified in U2OS-NBS1 and U2OS-53BP1 cells with the help of the dihydrorhodamine 123 test. To this end U2OS cells were kept on melting ice (0.8 °C) for 30 min. After treatment with hypothermia cells were harvested and dihydrorhodamine 123 (DHR, final concentration, 5 μM; Thermo-Fisher, D23806) was added as described by the producer. Cells were incubated with DHR for 30 min, washed with PBS and the level of fluorescence at 528 nm was measured with a LSR II flow cytometer (Becton Dickinson, USA). A computer system BD FACS DiVa (version 6.0, Becton Dickinson) was used for data acquisition and analysis. Data for 20,000 events were stored. A control test (keeping cells at 37 °C) and a positive control test with 3% and 5% H_2_O_2_ (percent values correspond to final concentration of H_2_O_2_ in the culture medium) were performed. Treatment with H_2_O_2_ lasted for 30 min at 37 °C. Three independent experiments with each cell line were carried out.

### Distribution of cells in the cell cycle

Following radiation exposure at the two temperatures, U2OS-53BP1 cultures were set up for the cell cycle distribution (Fig. 1C). Distribution of cells in the cell cycle were analysed by flow cytometry (Becton Dickinson FACSCaliburTM). Fixed cells were permeabilized and labelled with propidium iodide (PI) that stains DNA quantitatively. Results were analysed by ModFit LT™ (Verity, Software House) and the percentages of cells occupying different phases of the cell cycle were calculated.

### Clonogenic survival assay

Following radiation exposure U2OS-53BP1 cultures were set up for the clonogenic survival assay (Fig. 1D). Cells were plated in 6 cm diameter Petri dishes containing 5 ml of medium. Cells were seeded at a density of 100 cells/well in control panel and of 200 and 400 cells/well in the irradiated panel and next exposed to 2 Gy of gamma radiation as described above. Cells were exposed to hypothermia for 30 min before radiation. Following irradiation, cells were incubated for 14 days to allow colony formation. Colonies were stained with 5% Giemsa in a 25% methanol:water solution. Surviving fractions were calculated as described in^15^. Surviving fractions of irradiated cells were calculated with reference to plating efficiencies of control cells kept at the appropriate temperature.

### Micronucleus assay

Following radiation exposure U2OS-NBS1 and U2OS-53BP1 cultures were set up for the micronucleus (MN) assay (Fig. 1B). To this end cytochalasin B (final concentration, 5 mg/mL; Sigma-Aldrich) was added immediately after irradiation and cells were harvested at three time points post irradiation: 20 h, 26 h and 32 h. Cells were detached by trypsin before harvesting and fixed as described in Olofsson et al.^16^. The fixed cells were dropped on clean slides and stained with 10% Giemsa (Merck, Germany) on the next day. Binucleated cells were scored for the presence of micronuclei using a light microscope and 400 magnifications. Scoring was performed in a blinded manner, with an attempt to score 1000 binucleated cells per treatment. Three independent experiments with each cell line were carried out.

### Statistical analysis and area under curve

In order to compare the focus formation and decay curves, the data were fitted to a Gaussian dose response model and areas under the curve (AUC) were calculated. The advantage of calculating AUC is that its value represents the whole time-yield curve and is only weakly influenced by extreme data points on the curve. Moreover, two AUC values can easily be compared by a statistical test. All calculations were carried out using SigmaPlot 12.5 (Systat Software Inc., USA). The Nalimov outlier test was used to identify outlying frequencies of MN^17^. Results achieved from exposing cells at the two temperatures were compared by measuring the effect size as described in^18^ and by two-sided Student’s *t* test, carried out either for paired values or assuming unequal variance. Only the difference between radiation-induced and control NBS1 and 53BP1 foci in cells exposed to 2 Gy of gamma radiation at 0.8 °C and allowed to repair at 0.8 °C was analysed by one way ANOVA on ranks. The significance level for the *t* test and ANOVA was set to 0.05 except for analysis of spontaneous foci where it was set to 0.017 to account for multiple comparisons (3 hypotheses). The value of 0.017 results from applying the Bonferroni correction (0.05/3). The type of applied test is specified in figure legends. The rational for applying the effect size test is the criticism of interpreting experimental results solely based on statistical significance^19,20^. The effect size thresholds were assumed as non-existent (d value < 0.2), small (d value 0.2–0.5), medium (d value 0.5–0.8), large (d value 0.8–1.3) and very large (d value > 1.3). For Student’s *t* test, a p value of < 0.05 was considered significant. Where appropriate, results of both tests are reported.

## Results

A graphical summary of the performed experiments is given in Fig. 1.

### Kinetic of foci formation and decay after 37–37 and 0.8–37 exposure scenarios

**Figure 2:**
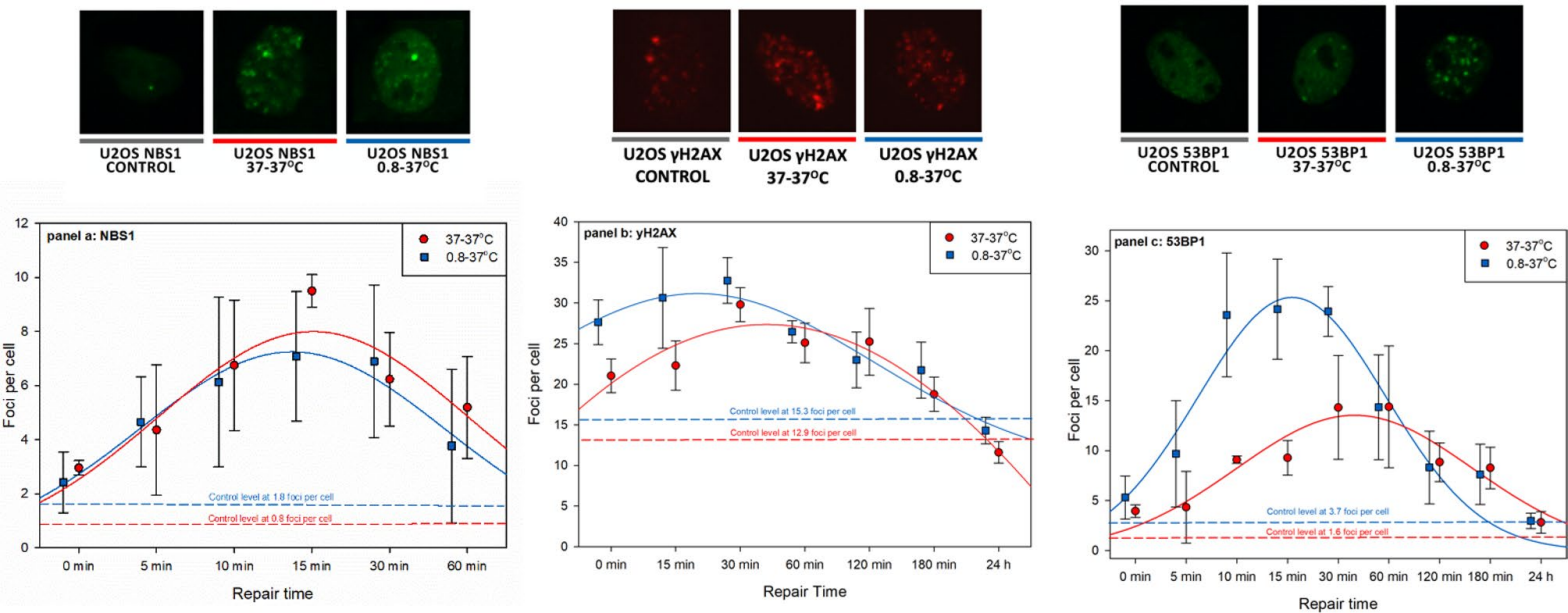
Impact of temperature at exposure: kinetics of NBS1 (**a**), γH2AX (**b**) and 53BP1 (**c**) foci formation and decay in U2OS cells exposed to 2 Gy of gamma radiation at 37 °C or 0.8 °C and allowed to repair at 37 °C. Time scale is not proportional. See Fig. 1A for an explanation of the treatment groups. Horizontal dashed lines represent control levels of foci. Error bars represent standard deviations from 3 experiments. AUC values were analysed by Cohen’s effect size test and by two-sided Student’s *t* test, assuming unequal variance. The results are described in the text. Images show representative nuclei with foci fixed 15 min post exposure. No counterstaining of nuclei was applied.

Results of the kinetic of NBS1, γH2AX and 53BP1 foci formation and decay in U2OS cells that were irradiated at 37 °C or 0.8 °C and allowed to repair at 37 °C (37–37 and 0.8–37 scenarios—see Fig. 1) are shown in Fig. 2. NBS1 foci were weak and hardly detectable after 60 min post exposure, so scoring was restricted to 0–60 min.

No strong impact of hypothermia was detected for NBS1 foci, although foci induced at 0.8 °C tended to decay faster than at 37 °C (Fig. 2a), resulting in higher mean AUC value in 37 °C irradiated cells. However, the difference between the AUC values was small (d = 0.29) and not significant (p = 0.74). The frequency of spontaneous foci was somewhat higher in cells cooled down (1.18 ± 0.80 foci per cell) than in those kept at 37 °C (0.82 ± 0.67 foci per cell) but the effect was medium (d = 0.5) and not significant (p = 0.57). In contrast, exposing cells at 0.8 °C lead to a stronger induction of γH2AX foci, but only during the first 30 min of repair (Fig. 2b). The difference between the AUC values was very large (d = 1.88) and significant (p = 0.033). Temperature at exposure had no impact on the kinetics of focus decay. The level of spontaneous foci was higher in cell cooled down (15.33 ± 1.77 foci per cell) than in those kept at 37 °C (12.87 ± 1.80 foci per cell) and the effect was very large (d = 1.38) but not significant (p = 0.66). At the level of 53BP1 foci, hypothermia had the strongest effect of all three focus types, but, similarly as for γH2AX foci, only during the first 30 min of repair time (Fig. 2c). At the peak of the kinetics curve, the focus frequency in cells exposed at 0.8 °C was 25 per cell as compared to 12.5 per cell in cells exposed at 37 °C. The difference between the AUC values was very large (d = 8.4) and significant (p = 0.033). Temperature at exposure had no impact on the kinetics of focus decay. Cooling cells down enhanced the level of spontaneous foci: the difference in frequency of spontaneous 53BP1 foci in cells cooled to 0.8 °C (3.68 ± 0.96 foci per cell) and those kept at 37 °C (1.59 ± 1.03 foci per cell) was very large (d = 2.1) and on the borderline of significance (p = 0.062).

A nested analysis of 53BP1 foci in cells exposed at 37 °C and at 0.8 °C and incubated at 37 °C for 15 min was carried where the size of nuclei was considered. The aim of the analysis was to check if the hypothermia effects was cell cycle phase dependent. Stratification of the data according to nucleus size allowed differentiating between S-phase and G_1_/G_2_ phase cells. The hypothermia effect was seen in both strata of nuclei suggesting that it is not cell cycle dependent. A detailed description of the analysis and results is given in the Supplementary Material 1.

### Kinetic of foci formation and decay after 0.8–0.8 exposure scenario

**Figure 3:**
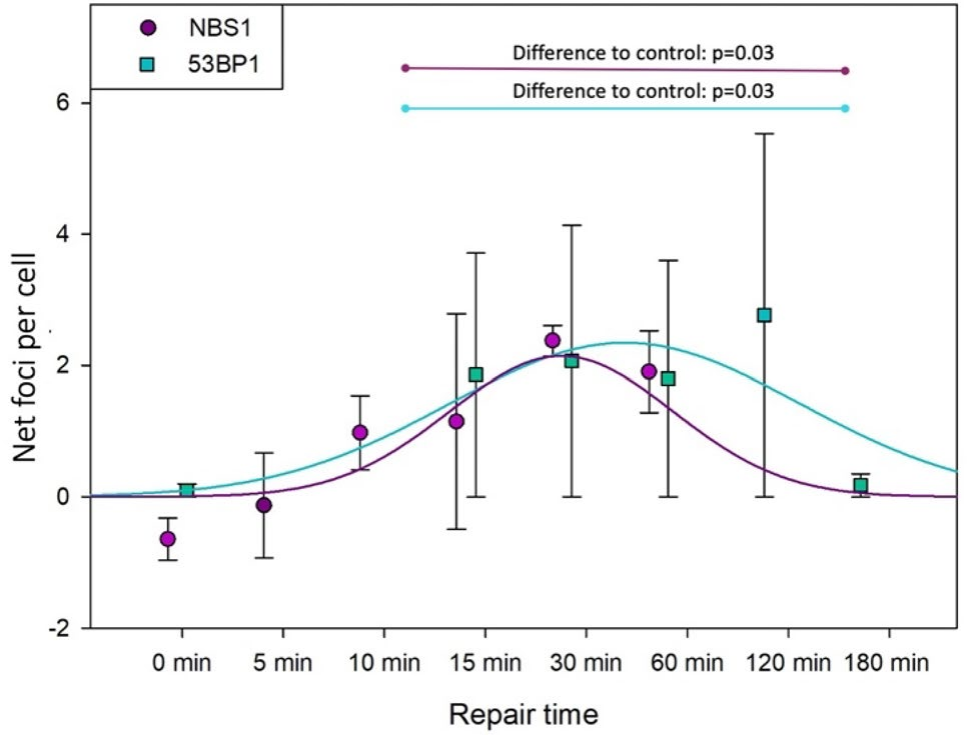
Repair at 0.8 °C: kinetics of NBS1 and 53BP1 foci formation and decay in U2OS cells exposed to 2 Gy of gamma radiation at 0.8 °C and allowed to repair at 0.8 °C. Shown are focus frequencies following subtraction of control values. Time scale is not proportional. See Fig. 1A for an explanation of the treatment groups. Blue dashed line represents the control value. Error bars represent standard deviations from 3 experiments. One way ANOVA on ranks was performed for data points corresponding to 10–180 min.

The net results (calculated by subtracting control values) of NBS1 and 53BP1 foci in cells exposed to 2 Gy and incubated at 0.8 °C (the 0.8–0.8 exposure scenario—see Fig. 1A) are shown in Fig. 3. The frequencies of the spontaneous foci are shown in Fig. 5 and discussed in the paragraph below. Because of differences in the formation kinetics of NBS1 and 53BP1 foci, the former was scored after 0, 5, 10, 15, 30 and 60 min and the latter after 0, 15, 30, 60, 120 and 180 min. No foci could be scored after 24 h post exposure because cells did not survive that long at 0.8 °C. Despite maintaining the cells at 0.8 °C both NBS1 and 53BP1 foci showed the typical kinetic of focus formation and decay demonstrating an ongoing DDR. However, the effect of radiation was weak and results from repeat experiments varied, leading to large standard deviations. For NBS1, the highest focus frequency was 2.4 (at 30 min post exposure) which is only slightly higher than the control. No radiation-induced NBS1 foci were observed after 0 and 5 min post exposure. For 53BP1, the highest focus frequency was 2.8 per cell (at 120 min post exposure) which is lower than the control value. No 53BP1 foci were observed at 0 min post exposure. The radiation-induced NBS1 and 53BP1 focus frequencies in the time range 10–180 min were significantly higher than the respective control. The mean AUC value of the net NBS1 AUC (6.12 ± 2.19) was somewhat lower than that of the mean this 53BP1 AUC (8.55 ± 4.94), this not being due to the maximal level of focus frequency, but to a slower decay of 53BP1 foci. However, no NBS1 foci were scored beyond 60 min, so a precise comparison of the decay kinetics is not possible, and a comparison of AUC values is not meaningful.

### Analysis of oxidative stress

**Figure 4:**
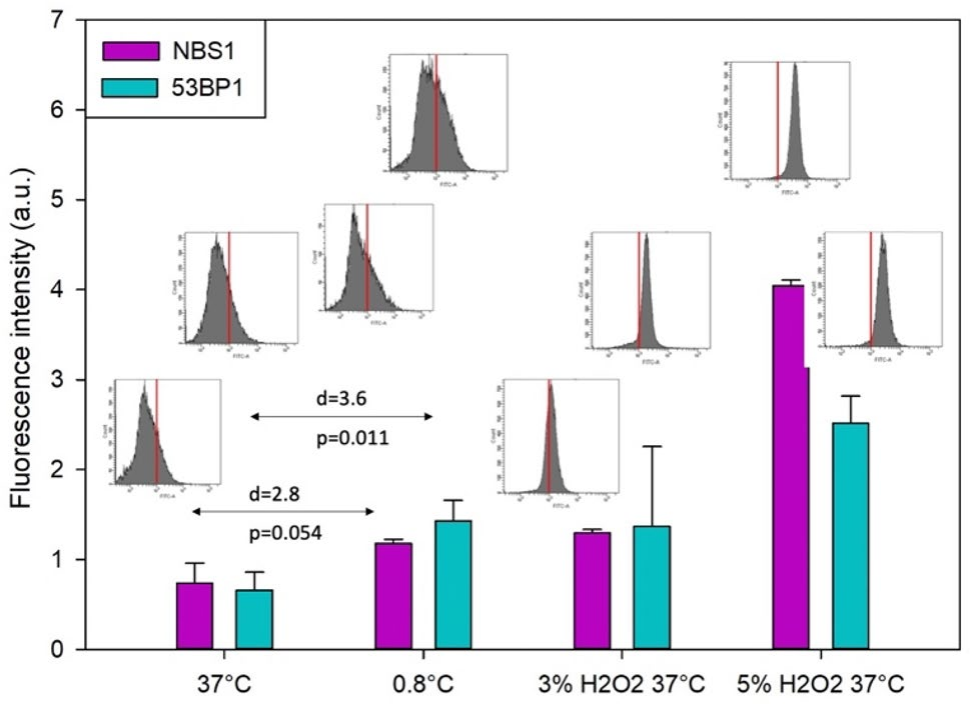
Oxidative stress in U2OS cells (levels of dihydrorhodamine 123) exposed to hypothermia and two concentrations of H_2_O_2_, *a.u.* arbitrary units. Results represent mean values from 3 experiments with U2OS-NBS1 and U2OS-53BP1 cells. Error bars represent standard deviations from 3 experiments. Histograms represent results from flow cytometric analyses. Red vertical lines mark the FITC-A level of 10^3^. D values represent Cohen’s d values, p values represent significance level from two-sided Student’s *t* test, assuming unequal variance. See Fig. 1E for exposure details.

The results of the oxidative stress test are shown in Fig. 4. The mean level of dihydrorhodamine 123 (shown as fluorescence intensity) in control cells kept at 37 °C was 739 ± 219 arbitrary units (a.u.) in U2OS-NBS1 cells and 661 ± 200 a.u. in U2OS-53BP1 cells. Keeping the cells on melting ice 0.8 °C for 30 min increased the mean signal intensity to 1181 ± 45 a.u. in U2OS-NBS1 cells and to 1432 ± 226 a.u. in U2OS-53BP1 cells. The impact of hypothermia was very large (d = 3.6 for 53BP1 and d = 2.8 for NBS1), the respective p values were 0.011 (significant) and 0.054 (borderline significant). In order to relate the hypothermia-induced levels of dihydrorhodamine 123 to a positive control, the effect of treating cells with 3% and 5% H_2_O_2_ for 30 min was assessed. 3% H_2_O_2_ resulted in a mean signal intensity of 1297 ± 37 a.u. and 1370 ± 888 a.u. in U2OS-NBS1 and U2OS-53BP1 cells, respectively. 5% H_2_O_2_ resulted in a mean signal intensity of 4044 ± 62 a.u. and 2517 ± 298 a.u. in U2OS-NBS1 and U2OS-53BP1 cells, respectively.

### Spontaneous foci 37–37, 0.8–37 and 0.8–0.8 exposure scenarios

**Figure 5:**
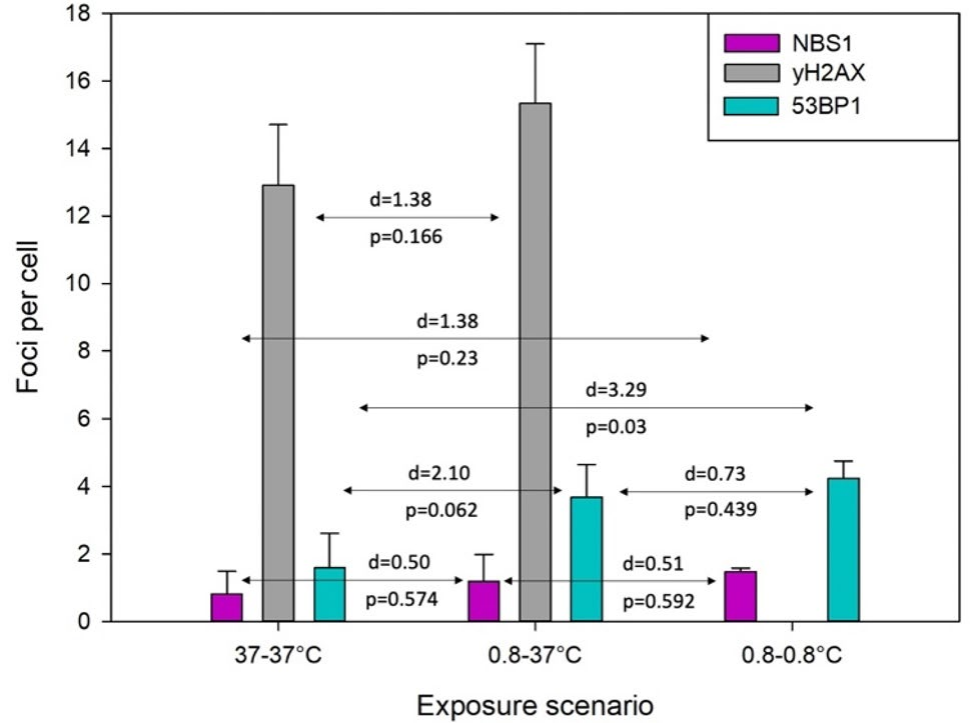
Frequencies of spontaneous foci in U2OS cells exposed to hypothermia without irradiation. Results represent mean values from 3 experiments with NBS1, 53BP1 and γH2AX foci. Error bars represent standard deviations from 3 experiments. D values represent Cohen’s d values. p values represent significance level from two-sided Student’s *t* test, assuming unequal variance. See Fig. 1A for explanation of the exposure scenarios.

The spontaneous frequency of NBS1 and 53BP1 foci in cells kept at the 0.8–0.8 scenario (Fig. 1A) was higher as compared to the 37–37 exposure scenario. The spontaneous frequency of NBS1 foci per cell was: 1.48 ± 0.10 at 0.8 °C and 0.82 ± 0.67 at 37 °C, the difference being very large (d = 1.38) but not significant (p = 0.23). The spontaneous frequency of 53BP1 foci per cell was: 4.24 ± 0.80 at 0.8–0.8 and 1.59 ± 1.03 at 37 °C, the difference being very large (d = 3.29) but not significant at the significance level of 0.017 (p = 0.03). Hypothermia at both exposure scenarios 0.8–0.8 and 0.8–37 had a much stronger effect on the frequency of spontaneous 53BP1 foci than on the frequency of spontaneous NBS1 foci. The spontaneous frequencies of γH2AX foci were several-fold higher than those of the other foci. Hypothermia induced a very large effect (d = 1.38) which was not significant (p = 0.166). In order to visualise these effects, the frequencies of spontaneous NBS1, γH2AX and 53BP1 foci in cells treated according to the different exposure scenarios (see Fig. 1 for explanation) are summarised in Fig. 5.

### Cell cycle kinetics and colony formation

**Figure 6:**
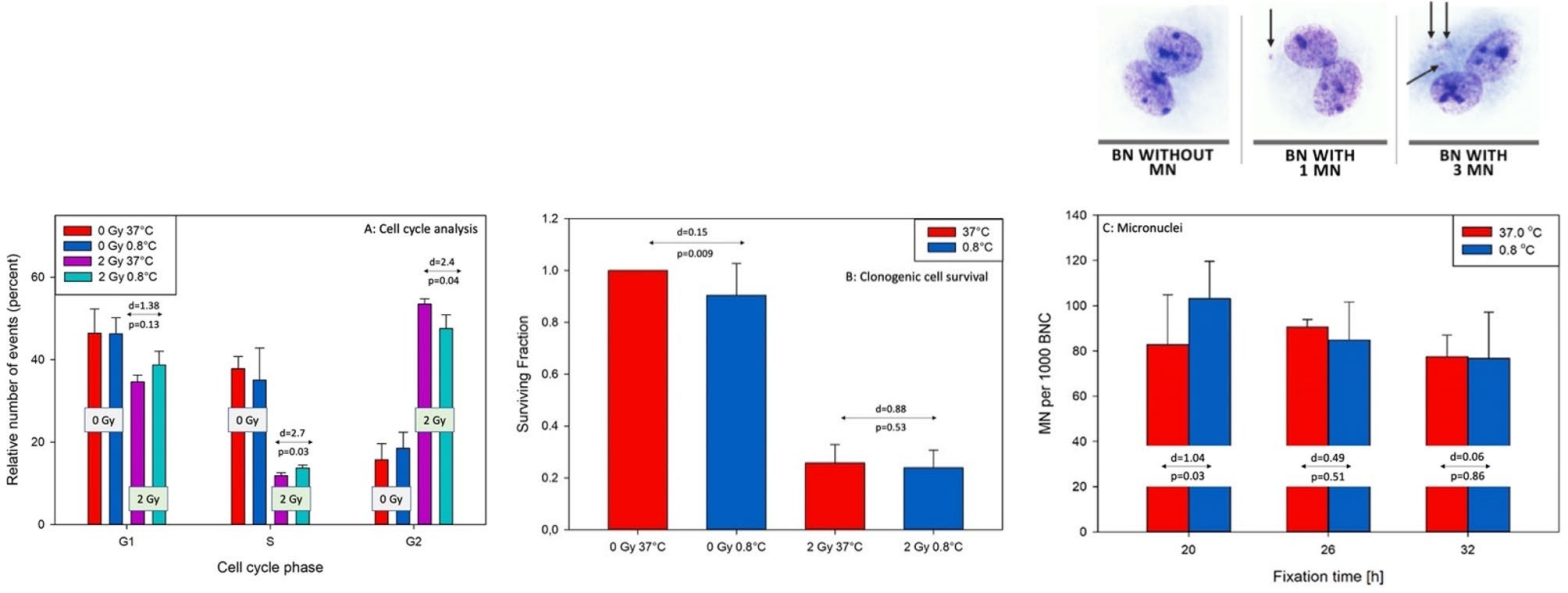
(**A**) distribution of cells in the cell cycle measured by flow cytometry, (**B**) clonogenic cell survival and (**C**) frequencies of micronuclei (MN) in U2OS cells irradiated with 2 Gy at 0.8 °C and 37 °C. See Fig. 1B–D for explanation of the exposure. In micronuclei test cells were harvested at three time points: 20 h, 26 h and 32 h of culture time, following addition of Cytochalasin B directly after irradiation. Error bars represent standard deviations from 3 repeats of cell cycle analyses, 12 repeats of clonogenic cells survival and 6 repeats of MN assay. D values represent Cohen’s d values. p values represent significance level from two-sided Student’s *t* test, assuming unequal variance. Arrows on images mark MN.

Analysis of cell cycle by flow cytometry revealed that in cells kept at 37 °C the distribution of cells in G_1_, S and G_2_ phases were, respectively, 46.5 ± 5,9%, 37.8 ± 3.0% and 15.7 ± 3.9% (Fig. 6A). A radiation dose of 2 Gy induced a distinct G_2_ cell cycle block resulting in a distribution of 34.6 ± 1.7% G_1_ cells, 11.9 ± 0.7% S cell and 53.5 ± 1.3% G_2_ cells. A 30 min hypothermia without radiation did not induce any detectable shift in the cell cycle distribution. However, a dose of 2 Gy delivered at 0.8 °C lead to increased numbers of cells in G_1_ (38.8 ± 3.3, d value = 1.38 and p = 0.13) and S (13.7 ± 0.7, d value = 2.7 and p = 0.03) and a reduction in the number of cells in G_2_ (47.6 ± 3.3, d value = 2.4 and p = 0.04).

Keeping cells for 30 min at 0.8 °C reduced the surviving fraction to 0.90 ± 0.12, being weak (d = 0.15) but significant (p = 0.009) (Fig. 6B). A dose of 2 Gy delivered at 37 °C reduced the surviving fraction to 0.25 ± 0.072 and when delivered at 0.8 °C to 0.24 ± 0.068. The difference between the 2 Gy-induced surviving fractions at the two temperatures was small (d = 0.88) and not significant (p = 0.53).

### Frequency of micronuclei

The spontaneous frequencies of MN did not differ between the cell lines and the temperature at exposure (MN per 1000 U2OS-NBS1 cells: 30 ± 5 at 0.8 °C and 21 ± 20 at 37 °C; U2OS-53BP1 cells: 27 ± 6 at 0.8 °C and 28 ± 5 at 37 °C; MN per 1000). Radiation-induced MN frequencies from both cell lines were pooled because, for a given temperature and fixation time, they did not differ (d value < 0.2, Cohen’s effect size test and p > 0.05). MN frequencies in U2OS-53BP1 cells harvested at 26 h in one experiment and in at 32 h in another experiment were identified as outliers by the Nalimov test and eliminated from the analyses. The results are summarised in Fig. 6C. A higher MN frequency was detected in cells exposed at 0.8 °C as compared to 37 °C and fixed at 20 h harvest time. The difference was large and significant (d = 1.04, p = 0.03). At 26 h fixation time an inverted result was seen, however its effect size was lower and not significant (d = 0.49, p = 0.51). No difference was seen at 32 h fixation time (d = 0.06, p = 0.86). Overall, the frequencies of MN tended to decrease with fixation time.

## Discussion

The aim of the study was to investigate the effect of hypothermia during ionising radiation exposure on the formation and decay of NBS1, γH2AX and 53BP1 foci in U2OS cells. In addition, the impact of hypothermia on cell cycle progression, clonogenic cell survival and the misrepair of DNA damage was investigated by analysing the frequency of micronuclei. The results show that hypothermia at irradiation, followed by recovery at 37 °C, lead to an increased formation of γH2AX and 53BP1 but not of NBS1 foci, as compared to irradiation at 37 °C. In cells irradiated and kept at 0.8 °C, 53BP1 and NBS1 foci formed and decayed, but at a level distinctly lower than at 37 °C. Finally, an attenuated G_2_ block, and enhanced frequencies of micronuclei were detected in cells exposed 0.8 °C (but only when harvested 20 h post irradiation). Hypothermia significantly reduced clonogenic cell survival of control, but not irradiated cells.

An observation which is relevant for the interpretation of the results was that hypothermia itself induced both NBS1, 53BP1 and γH2AX foci, the effect being particularly strong when NBS1 and 53BP1 foci were analysed in cells maintained at 0.8 °C. Also, it reduced clonogenic cell survival. It is known that hypothermia can induce oxidative stress in tissues of cooled organisms, but this effect is due to hypothermia-induced hypoxia and subsequent reperfusion, analogous to the process of ischemia and reperfusion^20^. This mechanism does not apply to our cell model system, but we hypothesized that cooling of U2OS cells to 0.8 °C for 30 min leads to reduced activity of enzymatic antioxidant systems^21^ or an impaired integrity of mitochondrial membranes, leading to release of reactive oxygen species (ROS). In order to check this, the level of oxidative stress was measured using the dihydrorhodamine 123 (DHR123) test which quantifies the nitrosative component of oxidative stress^22,23^. Keeping cells at 0.8 °C for 30 min lead to an increased level of oxidative stress the magnitude of which was equivalent to treating cells with 3% H_2_O_2_ for 30 min. It should be noted that hypothermia may induce oxidative stress selectively in U2OS cells, as we did not observe enhanced DNA damage response in PBL or TK6 cells exposed to 0.8 °C for 20 min^5,7^. Moreover, the hypoxia-induced oxidative stress was detectable as reduced clonogenic cell survival but was not strong enough to induce changes in cell cycle progression or MN. Further experiments are necessary to elucidate the cell-type specificity of the effect.

Irradiation of cells at 0.8 °C and allowing them to repair at 37 °C lead to a stronger induction of γH2AX and 53BP1 foci than when cells were irradiated at 37 °C. The effect cannot be accounted for by an increased level of ionisation damage at 0.8 °C because the level of ionisation events in an irradiated cell is determined by the absorbed dose and not the temperature at exposure. However, DNA damage induced by ionising radiation occurs via two mechanisms: through the direct interaction of radiation with the DNA molecule and indirectly, through radical species which are produced as consequence of water radiolysis^24^. Hypothermia has actually been shown to protect the DNA from the indirect action of radiation^6,25^ reducing the level of damage. Why then did we observe an augmented level of radiation-induced γH2AX and 53BP1 foci in cells exposed at 0.8 °C? Presently we do not know the answer but we speculate that it is an adaptive response to oxidative stress induced by hypothermia prior to irradiation. It is possible that the hypothermia-activated phosphorylation of γH2AX by ATM and the protein complex involving 53BP1^26^ over-reacts upon radiation-induced DNA damage at low temperature. Indeed, we have observed enhanced levels of phosphorylated ATM and DNA-PKcs in peripheral blood lymphocytes (PBL) irradiated at 0.8 °C^5^. However, hypothermia at exposure had a sparing effect on cytogenetic damage in PBL and did not lead to detectable changes of the formation and decay of γH2AX foci^27^, demonstrating cell-type specificity in the reaction of different cells to low temperature at exposure. With respect to U2OS cells it is interesting that the hypothermia effect on focus formation was not seen at the step of NBS1 foci formation, was mild at the step of H2AX foci and strong at the step of 53BP1 foci formation. The DDR to induced DSB proceeds in a chronological way: activation of NBS1 precedes the phosphorylation of H2AX that is followed by the activation of 53PB1^11^. The gradual development of the hypothermia effect suggests that it acts on signals downstream of NBS1 activation and increases stepwise. Currently, we do not know how long it lasts. Further investigations targeted at elucidating the mechanisms of the effect and its development downstream of 53BP1 activation are necessary and could include analyses of signalling pathways, activation of other proteins involved in DDR and application of radical scavengers in combination with hypothermia.

Higher frequencies of γH2AX as compared to 53BP1 foci were observed after 30 min post exposure in cells exposed under the 37–37 scenario. Published results show that both foci colocalise around this time point post irradiation^28^, so the difference detected by us suggests an underestimation of 53BP1 focus frequency. Here it should be recalled that γH2AX foci were detected with the help of immunostaining while 53BP1 foci were visualised thanks to GFP tagging. The signal strength of GFP is usually weaker than that of immunostaining^29^ so this could explain the difference.

As mentioned in the introduction, irradiation and maintaining of cells on melting ice is common practice to inhibit DNA damage repair in experiments aiming at studying the mechanisms of DSB induction and repair^28,30^. Our results with cells irradiated and maintained at 0.8 °C for 180 min post irradiation demonstrate that U20S cells sense and activate the DDR despite being kept on ice. The level of focus induction was much lower and more stretched out in time than when cells were allowed to repair at 37 °C. A similar weak activation of γH2AX foci in VH10 cells irradiated and kept on ice for 30 min was reported by Markova et al.^28^. The authors also used pulsed field gel electrophoresis to measure the development of DSB in cells kept on ice and did not notice any ongoing repair. It is possible that the induction of foci in cells kept at on ice represents a DDR step not followed by repair of DSB^31^. On the other hand, we observed a decay of 53BP1 foci after 180 min post exposure and if NBS1 foci 60 min post exposure in cells kept on ice, indicating downregulation of DDR signals, as if some damage was repaired. It would have been interesting to follow the development of foci for longer times, however, cells kept on melting ice for longer than 180 min started to detach and die (conclusion based on visual inspection) so longer analysis was not possible. An interesting question is how the decay of foci at 0.8 °C influences the process of transforming DNA damage into cytogenetic damage. Presently we do not know this but plan further experiments to investigate this question. In any case, the presented results demonstrate that DDR signalling is initiated and progresses in cells irradiated and kept on melting ice. Moreover, hypothermia itself induces some form of DNA damage. This fact should be borne in mind when planning studies on the mechanisms of DNA damage and repair.

In order to investigate the outcome of modified DNA damage repair in cells exposed at hyperthermia we analysed the distribution of cells in the cell cycle, clonogenic cells survival and frequency of micronuclei. Hyperthermia at exposure lead to a reduction of the radiation-induced G_2_ block and accumulation of cells in G_1_ and S. Attenuation of the G_2_ block was shown to sensitize cells to ionising radiation^32^ and our results support this: cells irradiated at 0.8 °C showed a higher frequency of MN, but only at the earliest fixation time. Why was the effect not seen in cells harvested at later time points? The rationale for analysing MN in binucleated cells harvested at different time points post irradiation (a method known as the multiple fixation regimen) is that, in cells irradiated during asynchronous growth, the analysis yields a more meaningful frequency of cytogenetic damage compared to a single fixation regimen^7,33^. Binucleated cells appearing at the earliest fixation time post exposure were towards the late end of the cell cycle at the time of exposure, characterized by a high relative radiosensitivity. The later the fixation time, the earlier in the cell cycle were the scored binucleated cells. Hence, scoring MN in binucleated cells harvested at multiple time points allows detecting cytogenetic damage induced in various phases of the cell cycle. The observation of a higher frequency of MN in cells exposed at 0.8 °C as compared to 37 °C at the earliest fixation time point is a consequence of the attenuated G_2_ block and reflects an increased level of misrepaired damage in cells that were approaching mitosis. We have carried out 6 replicate experiments and the difference was observed in 5 cases, suggesting that the effect is highly reproducible. At later fixation time points the MN frequencies in cells exposed at 0.8 °C and 37 °C did not differ suggesting that the radiosensitising effect of hypothermia in U2OS cells is restricted to late phase of the cell cycle. The effect is not strong as it did not modify clonogenic cell survival.

The results obtained in this study differ from those reported by us earlier in TK6 cells and PBL. In previous investigations we observed reduced frequencies of MN in TK6 cells exposed at 0.8 °C, but only at the first fixation in a multi fixation regimen^7^, a result which we interpreted as evidence for slowing down of the cell cycle by hypothermia. However, in a subsequent investigation with flow cytometry, no effect of hypothermia on the cell cycle could be detected^34^. The provided explanation was that that the temperature effect was either caused by selective elimination of cells damaged by radiation at 0.8 °C or by a shift in cell cycle progression that was too small to be detected at the level of flow cytometry. In PBL, the sparing effect of hypothermia was repeatedly observed at the level of radiation-induced aberrations and MN. The problem of heterogeneity of the target cell population with respect to radiosensitivity, as encountered when working with asynchronously growing cells, does not apply to PBL because, in peripheral blood, they are synchronised in the G_0_ phase of the cell cycle^35^. Indeed, we could show that reduced frequencies of MN are observed in all PBL irradiated at 0.8 °C and harvested according in a multiple fixation regimen^36^. Furthermore, we could exclude that it is due to a selective elimination of damaged cells by apoptosis^36^. We then used the technique of premature chromosome condensation to follow the early steps of transformation of DNA damage to chromosomal aberrations and concluded that hypothermia at exposure promotes the transformation. The conclusion was supported by the observation that radiation exposure at 0.8 °C is associated with augmented activation of the DNA damage response proteins ATM and DNA-PK^5^. However, no temperature effect could be seen at the level of γH2AX foci^27^. Obviously, the reaction of cells to hypothermia is cell type specific and the responsible mechanisms remain to be elucidated.

## Conclusions

Placing cells on melting ice induces DNA damage and influences the DNA damage response following exposure to ionizing radiation. This effect should be borne in mind when cooling cells on melting ice in order to inhibit DNA repair during the process of DNA damage induction.

## Supplementary Information

Supplementary Information.
