# Supplementary material for: Hypothermia differentially modulates the formation and decay of NBS1, γH2AX and 53BP1 foci in U2OS cells exposed to gamma radiation
Source: Sci Rep. 2022 Apr 7;12:5878. doi: 10.1038/s41598-022-09829-y (PMC8989987; doi:10.1038/s41598-022-09829-y)
Supplement: Supplementary file 1 — Supplementary Information. [file 41598_2022_9829_MOESM1_ESM.docx]

**Supplementary material 1**

53BP1 foci in nuclei of U2OS cells exposed in G1/G2 and S-phase of the cell cycle

**Aim**

The aim of this investigation was to compare the level of radiation-induced 53BP1 foci in cells in different positions in the cell cycle. The rational for the analysis was to test the possibility that the hypothermia effect will vary depending on factors such as chromatin condensation and DSB repair pathway that depend on the position of a cell in the cell cycle. The cell cycle position was assessed based on the nucleus size, estimated both as mean diameter and area. The analysis focused on cells exposed at 0.8°C and 37°C, incubated at 37°C and fixed after 15 minutes (scenarios 0.8-37 and 37-37, respectively), where the hypothermia effect was strongest (Figure 2C in the manuscript). Foci were scored manually on computer images.

The estimation of nucleus size is a very crude assessment of the position in the cell cycle but it is the only available one. Nuclei were not stained with any DNA dye because this would impair the scoring of foci (detected thanks to GFP-tagging of NBS1 and 53BP1). So the position of a cell in the cell cycle could not be determined based on measuring the DNA content of a nucleus.

Based on the nucleus size, cells can be assigned to S-phase (large nuclei) and G1+G2 phases (small nuclei). The assignment was based on the distribution of cells in the cell cycle.

**Materials and methods**

53BP1 foci per nucleus and the nucleus size were manually scored on screen images of U2OS cells exposed to 2 Gy of gamma radiation at 37°C or 0.8°C and allowed to repair at 37°C for 15 min. U2OS cell nuclei have round or oval shapes. The longest and shortest diameter of a nucleus was measured and used to calculate the mean diameter (average of two measurements) and the nucleus area (product of the two measurements). The nucleus diameters were measured with a ruler at a constant magnification of an image (100%).

The distribution of cells in the cell cycle was determined by flow cytometry and the results are shown in Figure 6A of the manuscript. They show that 40% of proliferating control cells are in the S-phase. This value was used for assigning cells on images to phases in the cell cycle, assuming that it applied to a cell population harvested 15 min after a dose of 2 Gy. Cells were sorted according to the nucleus size (separately based on the mean nucleus diameter and the nucleus area) and the frequency of foci was determined in 40% of cells with the largest nuclei (corresponding to S-phase) and 60% of cells with smallest nuclei (corresponding to G1 and G2-phase cells).

**Results and conclusions**

Summed results are shown in table S1 and raw results in S2. Figure S1 shows the results in a graphic form. A total of 110 cells were scored in the 37-37 arm and 106 the 0.8-37 arm. The number of S-phase cells was, respectively, 44 and 38. As evident from Figure 2C in the manuscript, the mean focus frequency in all cells was higher in the 0.8-37 arm (23.88±11.39) as compared to cells of the 37-37 arm (8.55 ± 8.56). Similar results were observed in cells stratified according to the cell cycle phase. The frequency of foci in G1/G2-phase cells of the 0.8-37 arm was 22.0±10.74 as compared to 9.9±7.49 in cells of the 37-37 arm. The frequency of foci in S-phase cells of the 0.8-37 arm was 27.2±11.77 as compared to 7.77±9.99 in cells of the 37-37 arm. In both cases the difference is large and significant. See article for a description of the applied statistical tests.

**In conclusion it can be stated that the hypothermia effect does not depend on the phase of the cell cycle, at least when S-phase and G1/G2 phase cells are compared.**

**Figure S1**. Box plots of 53BP1 foci in cells exposed to 2 Gy and analysed 15 min post exposure. For exposure scenarios see Figure 1 in the article. Horizontal line inside the boxes represent the median. Round symbol represent outliers. D values represent the effect size (Cohen´s test), p-values represent level of significance (t-test). Assignment of nuclei to a cell cycle phase was done based on the nucleus area.


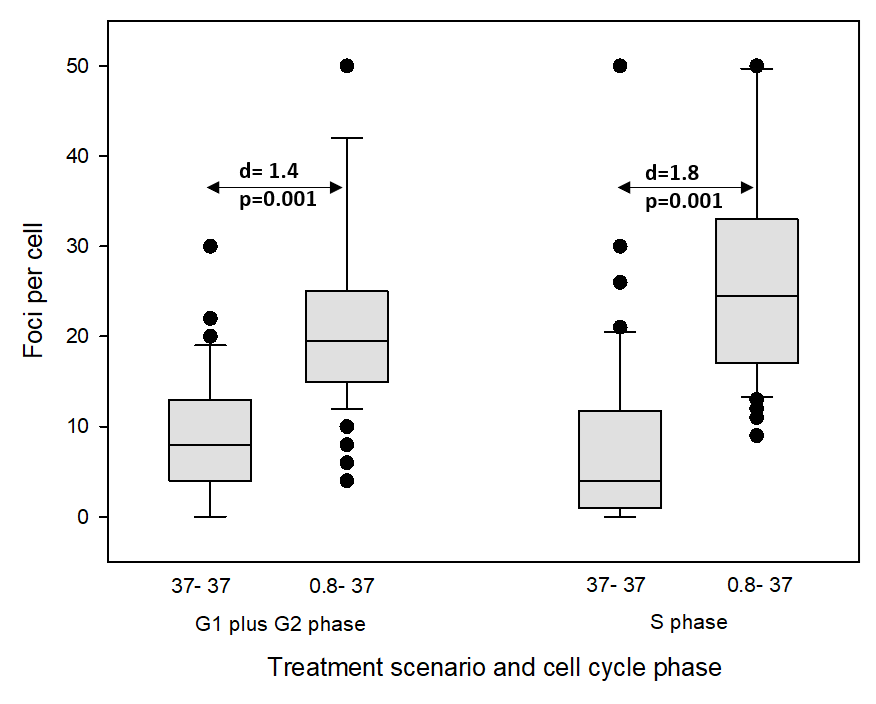


**Table S1A.** Tabulated results. N= number of analysed nuclei, std: standard deviation. For exposure scenarios see Figure 1 in the article. Nucleus diameters and nucleus areas are given in arbitrary units.

| **37-37 exposure scenario** | | | | | |  | **0.8-37 exposure scenario** | | | | | |
| --- | --- | --- | --- | --- | --- | --- | --- | --- | --- | --- | --- | --- |
| **Cycle phase** | **Measure-ment** | **Mean**  **nucleus**  **diameter** | **Foci per**  **nucleus** | **Nucleus**  **area** | **Foci per**  **nucleus** |  | **Cycle phase** | **Measure-ment** | **Mean**  **vucleus**  **diameter** | **Foci per nucleus** | **Nucleus area** | **Foci per nucleus** |
| **All** | N = 110 |  | | | |  | **All** | N=106 |  | | | |
|  | mean | 10.76 | 8.55 | 114.43 | 8.55 |  |  | mean | 10.88 | 23.88 | 118.09 | 23.88 |
|  | median | 11.00 | 7.00 | 113.50 | 7.00 |  |  | median | 10.50 | 22.00 | 110.00 | 22.00 |
|  | std | 1.78 | 8.56 | 38.45 | 8.56 |  |  | std | 1.66 | 11.39 | 38.02 | 11.39 |
| **G1/G2** | N=66 |  | | | |  | **G1/G2** | N=64 |  | | | |
|  | mean | 10 | 9 | 91 | 9 |  |  | mean | 10 | 22 | 95 | 22 |
|  | median | 10 | 8 | 93.5 | 8 |  |  | median | 10 | 19.5 | 99 | 19.5 |
|  | std | 1.15 | 7.58 | 21.44 | 7.49 |  |  | std | 0.74 | 10.74 | 15.06 | 10.74 |
| **S** | N=44 |  | | | |  | **S** | N=38 |  | | | |
|  | mean | 12.47 | 7.50 | 150.23 | 7.77 |  |  | mean | 12.63 | 27.20 | 156.95 | 27.20 |
|  | median | 12.25 | 4 | 141.5 | 4 |  |  | median | 12 | 25 | 143.5 | 25 |
|  | std | 1.06 | 9.84 | 29.45 | 9.99 |  |  | std | 1.24 | 11.77 | 32.06 | 11.77 |

**Table S1B.** Raw results. For exposure scenarios see Figure 1 in the article. Nucleus diameters and nucleus areas are given in arbitrary units.

| **37-37 exposure scenario** | | | | | |  | **0.8-37 exposure scenario** | | | | | |
| --- | --- | --- | --- | --- | --- | --- | --- | --- | --- | --- | --- | --- |
| **Cycle**  **phase** | **Nucleus**  **diameter** | **Foci per**  **nucleus** |  | **Nucleus**  **area** | **Foci per**  **nucleus** |  | **Cycle**  **phase** | **Nucleus**  **diameter** | **Foci per**  **nucleus** |  | **Nucleus**  **area** | **Foci per**  **nucleus** |
| **G1/G2 phase** | 6.5 | 7 |  | 42 | 7 |  | **G1/G2 phase** | 8 | 21 |  | 60 | 21 |
|  | 7 | 7 |  | 48 | 7 |  |  | 8 | 16 |  | 64 | 16 |
|  | 7.5 | 7 |  | 54 | 7 |  |  | 8.5 | 6 |  | 66 | 12 |
|  | 7.5 | 7 |  | 55 | 3 |  |  | 8.5 | 12 |  | 70 | 6 |
|  | 7.5 | 8 |  | 56 | 7 |  |  | 8.5 | 16 |  | 72 | 13 |
|  | 8 | 12 |  | 56 | 8 |  |  | 8.5 | 13 |  | 72 | 20 |
|  | 8 | 3 |  | 60 | 12 |  |  | 9 | 13 |  | 72 | 16 |
|  | 8 | 7 |  | 60 | 0 |  |  | 9 | 12 |  | 72 | 13 |
|  | 8 | 5 |  | 63 | 7 |  |  | 9 | 20 |  | 77 | 12 |
|  | 8.5 | 0 |  | 64 | 5 |  |  | 9 | 17 |  | 77 | 17 |
|  | 8.5 | 0 |  | 66 | 6 |  |  | 9 | 18 |  | 78 | 20 |
|  | 8.5 | 6 |  | 70 | 0 |  |  | 9 | 22 |  | 80 | 18 |
|  | 8.5 | 14 |  | 70 | 14 |  |  | 9 | 18 |  | 80 | 22 |
|  | 8.5 | 6 |  | 72 | 0 |  |  | 9 | 13 |  | 80 | 18 |
|  | 9 | 10 |  | 72 | 2 |  |  | 9.5 | 15 |  | 80 | 13 |
|  | 9 | 0 |  | 72 | 6 |  |  | 9.5 | 26 |  | 84 | 15 |
|  | 9 | 0 |  | 72 | 1 |  |  | 9.5 | 8 |  | 84 | 18 |
|  | 9 | 2 |  | 75 | 8 |  |  | 9.5 | 20 |  | 84 | 15 |
|  | 9 | 20 |  | 77 | 20 |  |  | 9.5 | 25 |  | 84 | 22 |
|  | 9 | 16 |  | 77 | 4 |  |  | 9.5 | 15 |  | 84 | 50 |
|  | 9 | 1 |  | 80 | 16 |  |  | 9.5 | 18 |  | 88 | 26 |
|  | 9 | 5 |  | 80 | 11 |  |  | 9.5 | 22 |  | 88 | 25 |
|  | 9 | 1 |  | 81 | 10 |  |  | 9.5 | 50 |  | 88 | 21 |
|  | 9 | 11 |  | 81 | 0 |  |  | 9.5 | 18 |  | 88 | 28 |
|  | 9 | 4 |  | 81 | 1 |  |  | 9.5 | 21 |  | 90 | 15 |
|  | 9.5 | 4 |  | 81 | 5 |  |  | 9.5 | 28 |  | 90 | 8 |
|  | 9.5 | 30 |  | 84 | 19 |  |  | 10 | 16 |  | 90 | 18 |
|  | 9.5 | 19 |  | 88 | 8 |  |  | 10 | 13 |  | 91 | 22 |
|  | 9.5 | 8 |  | 90 | 4 |  |  | 10 | 4 |  | 96 | 13 |
|  | 9.5 | 18 |  | 90 | 30 |  |  | 10 | 24 |  | 96 | 19 |
|  | 10 | 0 |  | 90 | 18 |  |  | 10 | 15 |  | 96 | 17 |
|  | 10 | 30 |  | 91 | 13 |  |  | 10 | 19 |  | 99 | 16 |
|  | 10 | 8 |  | 91 | 13 |  |  | 10 | 17 |  | 99 | 50 |
|  | 10 | 13 |  | 96 | 22 |  |  | 10 | 22 |  | 99 | 30 |
|  | 10 | 13 |  | 96 | 9 |  |  | 10 | 50 |  | 99 | 20 |
|  | 10 | 17 |  | 96 | 8 |  |  | 10 | 50 |  | 99 | 23 |
|  | 10 | 9 |  | 96 | 5 |  |  | 10 | 30 |  | 99 | 22 |
|  | 10 | 8 |  | 99 | 0 |  |  | 10 | 22 |  | 99 | 15 |
|  | 10 | 9 |  | 99 | 30 |  |  | 10 | 20 |  | 99 | 23 |
|  | 10 | 8 |  | 99 | 17 |  |  | 10 | 23 |  | 99 | 29 |
|  | 10 | 4 |  | 99 | 9 |  |  | 10 | 22 |  | 100 | 4 |
|  | 10 | 5 |  | 99 | 8 |  |  | 10 | 34 |  | 100 | 24 |
|  | 10.5 | 4 |  | 100 | 4 |  |  | 10 | 20 |  | 100 | 50 |
|  | 10.5 | 30 |  | 104 | 4 |  |  | 10 | 15 |  | 100 | 22 |
|  | 10.5 | 0 |  | 105 | 13 |  |  | 10 | 23 |  | 100 | 34 |
|  | 10.5 | 0 |  | 108 | 0 |  |  | 10 | 25 |  | 100 | 20 |
|  | 10.5 | 12 |  | 108 | 12 |  |  | 10 | 29 |  | 100 | 25 |
|  | 10.5 | 16 |  | 108 | 8 |  |  | 10.5 | 28 |  | 104 | 12 |
|  | 10.5 | 10 |  | 108 | 1 |  |  | 10.5 | 12 |  | 108 | 28 |
|  | 10.5 | 8 |  | 110 | 30 |  |  | 10.5 | 16 |  | 108 | 50 |
|  | 10.5 | 8 |  | 110 | 0 |  |  | 10.5 | 10 |  | 108 | 27 |
|  | 10.5 | 18 |  | 110 | 16 |  |  | 10.5 | 17 |  | 110 | 16 |
|  | 10.5 | 1 |  | 110 | 10 |  |  | 10.5 | 50 |  | 110 | 10 |
|  | 11 | 19 |  | 110 | 8 |  |  | 10.5 | 50 |  | 110 | 17 |
|  | 11 | 2 |  | 110 | 18 |  |  | 10.5 | 15 |  | 110 | 50 |
|  | 11 | 0 |  | 117 | 19 |  |  | 10.5 | 18 |  | 110 | 15 |
|  | 11 | 22 |  | 117 | 2 |  |  | 10.5 | 26 |  | 110 | 18 |
|  | 11 | 18 |  | 117 | 0 |  |  | 10.5 | 27 |  | 110 | 26 |
|  | 11 | 11 |  | 117 | 18 |  |  | 10.5 | 18 |  | 110 | 18 |
|  | 11 | 4 |  | 120 | 13 |  |  | 10.5 | 33 |  | 110 | 33 |
|  | 11 | 4 |  | 120 | 2 |  |  | 11 | 25 |  | 112 | 50 |
|  | 11 | 15 |  | 120 | 6 |  |  | 11 | 15 |  | 117 | 25 |
|  | 11 | 8 |  | 120 | 11 |  |  | 11 | 14 |  | 120 | 14 |
|  | 11 | 4 |  | 120 | 4 |  |  | 11 | 19 |  | 120 | 19 |
|  | 11 | 21 |  | 120 | 4 |  | **S phase** | 11 | 24 |  | 120 | 24 |
|  | 11 | 13 |  | 120 | 15 |  |  | 11 | 50 |  | 121 | 15 |
|  | 11 | 0 |  | 120 | 8 |  |  | 11.5 | 11 |  | 126 | 14 |
|  | 11 | 7 |  | 120 | 21 |  |  | 11.5 | 14 |  | 126 | 25 |
|  | 11.5 | 13 |  | 120 | 4 |  |  | 11.5 | 33 |  | 130 | 11 |
|  | 11.5 | 50 |  | 120 | 0 |  |  | 11.5 | 23 |  | 130 | 33 |
|  | 11.5 | 2 |  | 121 | 4 |  |  | 11.5 | 15 |  | 132 | 23 |
|  | 11.5 | 1 |  | 121 | 7 |  |  | 11.5 | 25 |  | 132 | 15 |
|  | 11.5 | 13 |  | 126 | 4 |  |  | 11.5 | 22 |  | 132 | 22 |
|  | 11.5 | 12 |  | 126 | 12 |  |  | 11.5 | 24 |  | 132 | 24 |
|  | 11.5 | 4 |  | 126 | 2 |  |  | 11.5 | 25 |  | 132 | 25 |
|  | 11.5 | 0 |  | 128 | 4 |  |  | 11.5 | 22 |  | 132 | 22 |
|  | 11.5 | 17 |  | 130 | 50 |  |  | 11.5 | 30 |  | 132 | 30 |
|  | 11.5 | 2 |  | 130 | 1 |  |  | 12 | 23 |  | 135 | 23 |
|  | 11.5 | 18 |  | 130 | 13 |  |  | 12 | 9 |  | 135 | 17 |
|  | 12 | 1 |  | 130 | 18 |  |  | 12 | 21 |  | 140 | 23 |
|  | 12 | 3 |  | 132 | 0 |  |  | 12 | 31 |  | 140 | 9 |
|  | 12 | 4 |  | 132 | 17 |  |  | 12 | 23 |  | 140 | 31 |
|  | 12 | 10 |  | 135 | 1 |  |  | 12 | 17 |  | 140 | 21 |
|  | 12 | 8 |  | 135 | 3 |  |  | 12 | 21 |  | 140 | 30 |
|  | 12 | 3 |  | 135 | 3 |  |  | 12 | 30 |  | 140 | 37 |
|  | 12 | 3 |  | 140 | 10 |  |  | 12 | 30 |  | 143 | 21 |
|  | 12 | 5 |  | 140 | 3 |  |  | 12 | 37 |  | 144 | 14 |
|  | 12 | 0 |  | 140 | 5 |  |  | 12.5 | 14 |  | 144 | 30 |
|  | 12.5 | 1 |  | 143 | 8 |  |  | 12.5 | 12 |  | 150 | 50 |
|  | 12.5 | 20 |  | 144 | 0 |  |  | 12.5 | 28 |  | 150 | 19 |
|  | 12.5 | 4 |  | 150 | 1 |  |  | 12.5 | 50 |  | 154 | 28 |
|  | 12.5 | 2 |  | 150 | 20 |  |  | 12.5 | 19 |  | 156 | 12 |
|  | 12.5 | 30 |  | 152 | 4 |  |  | 13 | 17 |  | 160 | 50 |
|  | 12.5 | 0 |  | 154 | 2 |  |  | 13 | 49 |  | 165 | 49 |
|  | 13 | 26 |  | 156 | 30 |  |  | 13 | 27 |  | 165 | 27 |
|  | 13 | 0 |  | 156 | 0 |  |  | 13 | 43 |  | 165 | 43 |
|  | 13 | 0 |  | 160 | 16 |  |  | 13 | 50 |  | 168 | 17 |
|  | 13 | 4 |  | 160 | 0 |  |  | 13.5 | 25 |  | 176 | 25 |
|  | 13 | 6 |  | 165 | 4 |  |  | 13.5 | 50 |  | 180 | 50 |
|  | 13 | 16 |  | 168 | 26 |  |  | 13.5 | 50 |  | 182 | 50 |
|  | 13 | 0 |  | 168 | 0 |  |  | 14 | 13 |  | 195 | 13 |
|  | 13 | 2 |  | 169 | 0 |  |  | 14.5 | 16 |  | 208 | 16 |
|  | 13.5 | 4 |  | 169 | 2 |  |  | 15 | 37 |  | 220 | 33 |
|  | 13.5 | 2 |  | 180 | 9 |  |  | 15.5 | 33 |  | 224 | 37 |
|  | 13.5 | 9 |  | 180 | 11 |  |  | 15.5 | 29 |  | 228 | 29 |
|  | 13.5 | 11 |  | 182 | 2 |  |  | 16 | 40 |  | 255 | 40 |
|  | 14 | 14 |  | 192 | 14 |  |  | | | | | |
|  | 14 | 0 |  | 195 | 0 |  |  |  |  |  |  |  |
|  | 14.5 | 0 |  | 210 | 0 |  |  |  |  |  |  |  |
|  | 16.5 | 3 |  | 270 | 3 |  |  |  |  |  |  |  |
